# Bällchen participates in proliferation control and prevents the differentiation of *Drosophila melanogaster* neuronal stem cells

**DOI:** 10.1242/bio.20148631

**Published:** 2014-09-04

**Authors:** Toma Yakulov, Ufuk Günesdogan, Herbert Jäckle, Alf Herzig

**Affiliations:** Max-Planck-Institut für biophysikalische Chemie, Abteilung Molekulare Entwicklungsbiologie, Am Fassberg 11, 37077 Göttingen, Germany; 1 Present address: Renal Division, University Hospital Freiburg, Hugstetter Strasse 55, 79106 Freiburg, Germany.; 2 Present address: Wellcome Trust/Cancer Research UK Gurdon Institute, University of Cambridge, Tennis Court Road, Cambridge CB2 1QN, UK.; 3 Present address: Max-Planck-Institut für Infektionsbiologie, Department Cellular Microbiology, Charitéplatz 1, 10117 Berlin, Germany.

**Keywords:** Bällchen, *Drosophila*, Neuroblasts, Stem cells

## Abstract

Stem cells continuously generate differentiating daughter cells and are essential for tissue homeostasis and development. Their capacity to self-renew as undifferentiated and actively dividing cells is controlled by either external signals from a cellular environment, the stem cell niche, or asymmetric distribution of cell fate determinants during cell division. Here we report that the protein kinase Bällchen (BALL) is required to prevent differentiation as well as to maintain normal proliferation of neuronal stem cells of *Drosophila melanogaster*, called neuroblasts. Our results show that the brains of *ball* mutant larvae are severely reduced in size, which is caused by a reduced proliferation rate of the neuroblasts. Moreover, *ball* mutant neuroblasts gradually lose the expression of the neuroblast determinants Miranda and aPKC, suggesting their premature differentiation. Our results indicate that BALL represents a novel cell intrinsic factor with a dual function regulating the proliferative capacity and the differentiation status of neuronal stem cells during development.

## INTRODUCTION

Multicellular organisms have to maintain a balance between cell proliferation and differentiation. Differentiation leads to mitotically quiescent cells, whereas development, growth, tissue homeostasis and regeneration require cellular proliferation (Buttitta and Edgar, 2007). One strategy to ensure a balance between these cellular processes is based on self-renewing stem cells. Stem cells are maintained as proliferative and undifferentiated cells, whereas their daughter cells initiate differentiation (Kim and Hirth, 2009). Therefore, stem cell divisions require repetitive cell fate decisions, which are either controlled by external signals that emanate from stem cell niches or dependent on asymmetrically distributed factors in the dividing stem cells (Kim and Hirth, 2009).

Neuronal stem cells of *Drosophila melanogaster*, called neuroblasts (NBs), represent a well-studied stem cell system, which depends on asymmetric distribution of cell fate determinants (Chia et al., 2008; Kim and Hirth, 2009; Knoblich, 2010). During embryogenesis, NBs delaminate from a neuroepithelium and maintain the apical-basal polarity of this epithelium. This inherited asymmetry is then used to localize cell fate determinants either at the apical or basal cell cortex of the NB, which subsequently leads to an asymmetric partitioning of these determinants between the two daughter cells during cell division (Knoblich, 2010). Self-renewed NBs inherit the apical cortical proteins such as the atypical protein kinase C, Par6, Bazooka/Par3, Inscuteable, Partner of Inscuteable and GαI, whereas the basal cortical proteins, that include cell fate determinants such as Prospero and Brat, are inherited by the differentiating ganglion mother cell (GMC). Their localized retention requires the coiled-coil adaptor protein Miranda (MIRA), which is subsequently degraded in the differentiating GMC (Shen et al., 1997). NB divisions are also characterized by a morphological asymmetry, since the differentiating ganglion mother cell (GMC) is much smaller than the self-renewed NB. In many NB cell lineages GMCs undergo only one further division to either generate a pair of neurons or glial cells that undergo terminal differentiation.

In other *Drosophila* stem cell systems, like the germline stem cells (GSCs), cell fate distinction is mainly mediated by extracellular signaling from a stem cell niche (Morrison and Spradling, 2008). We have recently found that GSC self-renewal requires the activity of the gene *bällchen* (*ball*) (Herzig et al., 2014), which encodes a member of the metazoan specific VRK-1 protein kinase family (Aihara et al., 2004). *ball* orthologous from vertebrates and invertebrates encode proteins that phosphorylate the Barrier-to-Autointegration Factor protein (BAF), which is proposed to participate in the establishment of higher order chromatin structures (Gorjánácz et al., 2007; Nichols et al., 2006; Bengtsson and Wilson, 2006; Lancaster et al., 2007). However, phenotypic analyses of *Drosophila ball* mutants showed that severe chromatin defects are restricted to the oocyte nucleus (Ivanovska et al., 2005). Notably, *ball* mutants show extensive degeneration of tissues that rely on the proliferation of undifferentiated progenitor cells or stem cells, such as the nervous system, the imaginal discs as well as the gonads (Cullen et al., 2005; Herzig et al., 2014), which suggests that *ball* has a role in the maintenance of progenitor and stem cells.

A central question in stem cell biology is whether mechanisms exist that maintain the undifferentiated state of cells irrespective of the mode by which these different stem cell populations establish their cell fate decisions during self-renewal. There is mounting evidence that the differentiation of stem cell descendants requires a lowering of their capacity to proliferate through down-regulation of growth related processes (Chia et al., 2008; Knoblich, 2010). Consistently, recent work comparing the transcriptomes of purified pNBs and differentiated neurons revealed that genes coding for components of metabolic pathways and ribosome biosynthesis were up-regulated in pNBs (Berger et al., 2012). To restrict proliferation and to allow differentiation of GMCs, ribosome biogenesis needs to be down-regulated in GMCs through the expression of the Brat protein (Bowman et al., 2008). However, it remained unclear to what extent the proliferative potential of stem cells is a prerequisite to maintain their undifferentiated state and thereby their capacity to self-renew. Here we report that the BALL kinase is required to maintain the proliferative potential of NBs and that this function of BALL is a prerequisite for self-renewal. Our results show that *ball* mutant NBs proliferate at a reduced speed and progressively lose stem cell markers and differentiate untimely during development. Recently, we reported that BALL is crucial to maintain the undifferentiated state of niche supported germline stem cells (Herzig et al., 2014). Therefore our results on neuronal stem cells indicate that distinct stem cell populations employ a common factor, BALL, to remain undifferentiated.

## MATERIALS AND METHODS

### Fly strains

Unless otherwise stated, all chromosomes and insertions are described in the Flybase database (http://flybase.org). The *ru ca e ball^2^* chromosome was generated by imprecise excision of the P{*EP*}*ball^EP863^* P-element integration after recombination to the recessive markers. The deletion associated with the *ball^2^* allele removes the *ball* initiation codon and parts of the kinase domain coding sequence. The chromosomes P{*neoFRT*}82B and P{*neoFRT*}82B *e ball^2^* were constructed by meiotic recombination. The transgene P{*w^+mC^ UASp-ball.T:Avic/EGFP = pballE*}2.1 contains the complete *ball* coding sequence for GAL4 dependent expression of a BALL-EGFP fusion protein with the P{*wor.GAL4.A*} neuroblast driver line (gift from J. Knoblich). Strains to identify *ball^2^* mutant animals were w^*^; ru ca e ball^2^/TM3, Sb^1^, P{35UZ}2 (embryos) and w^*^; ru ca e ball^2^/TM3, Ser^1^, P{ActGFP}JMR2 (larvae). For MARCM (Lee and Luo, 2001) we used *y*^1^ *w*^1118^ P{*70FLP*}3F/w^*^ P*{UAS-lacZ.p}*; P*{tubP-Gal4}*/+; P*{neoFRT}*82B P*{tubP-Gal80}*LL3/P*{neoFRT}*82B *e ball^2^* (*ball^2^* mutant clones), *y*^1^ *w*^1118^ P{*70FLP*}3F/w^*^ P*{UAS-lacZ.p}*; P*{tubP-Gal4}*/+; P*{neoFRT}*82B P*{tubP-Gal80}*LL3/P*{neoFRT}*82B (control wild type clones) and *y*^1^ *w*^1118^ P{*70FLP*}3F/w^*^ P*{UAS-lacZ.p}*; P*{tubP-Gal4}*/P*{ pballE*}2.1; P*{neoFRT}*82B P*{tubP-Gal80}*LL3/P*{neoFRT}*82B *e ball^2^* (rescued *ball^2^* mutant clones).

### Larval brain preparation

Staged larvae were obtained by collecting newly hatched larvae over 2 h intervals and placing them into food vials at controlled density. Optionally, placing vials in a 38°C water bath for 1 h induced *flipase* expression for generation of genetic mosaics (Lee and Luo, 2001). At indicated time points, the larval tissue was dissected from larvae in Schneider's cell culture medium (Life Technologies, Paisley, UK) within a 30 min interval before fixation.

### Antibody staining

Antibody incubations were done in PBS, 0.1% Triton X-100, 10% goat serum (PBTS) either over night at 4°C or for 2 h at room temperature. Washings between the incubations were two rinses in PBS, 0.1% Triton X-100 (PBT), followed by three changes in PBT for 20 min each. For immunofluorescence, embryos were fixed 20 min in 4% paraformaldehyde, PBS, 50 mM EGTA, pH 7.0, devitillinized, dehydrated in methanol and rehydrated (Rothwell and Sullivan, 2000). All other tissue was fixed for 10 min in the same solution. Blocking was done by a 20 min incubation in PBTS. Primary antibodies and dilutions were: affinity purified rabbit anti-BALL (1:400), rabbit anti-histone H3 S10ph (1:1000, Upstate Biotechnology, Lake Placid, NY), rabbit anti-aPKC (1:1000, Santa Cruz Biotechnology, Santa Cruz, CA), rabbit anti-Cleaved Caspase 3 (1:500, Cell Signaling Technologies, Boston, MA), rabbit anti-MIRA (1:1000, gift from J. Knoblich, IMBA, Vienna, Austria), chicken anti-beta Galactosidase (1:1000, Abcam, Cambridge, UK), mouse anti-PROS (1:25, DSHB, University of Iowa, Iowa City, USA), mouse anti-ELAV (1: 1:25, DSHB), mouse anti-REPO (1:25, DSHB), mouse anti-GRH (1:2, gift from Sarah Bray, University of Cambridge, Cambridge, UK). Secondary antibodies were: goat anti-rabbit or goat anti-mouse coupled to Alexa488, Alexa568 or Alexa635 (Life Technologies) and goat anti-chicken coupled to Cy2 (Jackson ImmunoResearch, Newmarket, UK). All secondary antibodies were used at a 1:500 dilution. For DNA staining, samples were incubated in PBT with 1 mg/ml RNase A for 10 min and stained for 10 min in PBT with 1 µg/ml Propidium iodide (Life Technologies) or 5 µM DRAQ5 (Biostatus, Shepshed, UK). After a single wash in PBT samples were embedded in ProLong Gold (Life Technologies).

### *In situ* hybridization

Embryos were fixed with 7.4% paraformaldehyde for 20 min and hybridized at 57°C using standard protocols. Digoxegenin (DIG) labeled RNA probes for *in situ* hybridizations were obtained from *ball* cDNA LD27410 (Source BioScience, Nottingham, UK). For non-fluorescent detection sheep anti-DIG-AP Fab (Roche, Mannheim, Germany) was used at 1:2000 in combination with the NBT/BCIP detection reagent (Roche) at 1:100 following manufacturer's instructions. For fluorescent detection, primary incubation with 1:2000 sheep anti-DIG (Roche) was followed by incubation with 1:1000 donkey anti-Sheep Biotin (Jackson ImmunoResearch). For signal amplification, embryos were incubated for 45 min with ABC reagents (Vector Laboratories, Peterborough, UK), followed by 5 minutes incubation with TSA Cyanine3 reagents (Perkin Elmer, Waltham, MA) diluted 1:50 following the manufacturer's instructions. Fluorescent RNA detection was then followed by antibody staining to detect proteins.

### Image analysis

Images were acquired on a Leica SP2 LSM or a Leica SP5 LSM. For quantitative analyses, z-stacks were sampled at 0.1 µm z intervals. Cell numbers were analyzed in Image J by manual markup of individual cells in the stacks (Cell Counter plugin). Volume analysis was carried out with a modified Connected Threshold Grower plugin and manual thresholding. Details on the modified plugin are available on request.

## RESULTS

### *ball* expression is enriched in neuronal stem cells

**Fig. 1.**
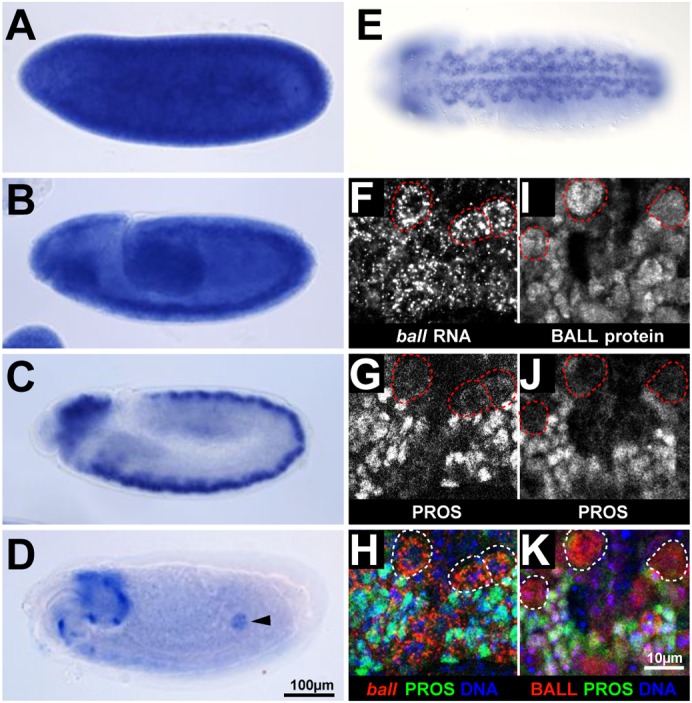
Expression of *ball* in embryonic neuroblasts. (A–E) *ball* mRNA expression detected by *in situ* hybridization of whole mount embryos. *ball* mRNA is contributed maternally to cleavage division stage embryos (A) and ubiquitously expressed up to stage 10 of embryogenesis (B). *ball* mRNA is enriched in the nervous system starting at stage 11 (C). In late embryos, *ball* mRNA staining fades in the nervous system (D) and becomes visible in the gonads (arrowhead in D). Orientation of embryos (A–D) is anterior to the left, dorsal side up. (E) *ball* mRNA is expressed in neuroblasts, which are arranged in a stereotyped pattern along the ventral nerve cord of stage 11 embryos. Enlarged ventral region of the embryo is shown; anterior region is up. (F–K) *ball* mRNA and Prospero (PROS) protein localization in the ventral nerve cord of a stage 11 embryo. *ball* mRNA expression is high in the large neuroblast cells (dashed circles) and low in the PROS expressing GMCs. The overlay shows the DNA channel to better visualize the cells (H). Enlarged ventral region of the embryo is shown; anterior region is up. Scale bar in A–E, 100 µm. Scale bar in F–K, 10 µm.

We analyzed the expression of *ball* by RNA *in situ* hybridization in embryos. During early syncytial cleavage divisions of the embryo and up to stage 10 of embryogenesis when most cells of the embryo are mitotically active, *ball* is expressed ubiquitously (Fig. 1A,B). From stage 11 onwards, however, *ball* transcripts become enriched in the nervous system, which is at this stage the major site of cell proliferation in the embryo (Fig. 1C). By the end of embryogenesis, *ball* transcripts fade from the mitotically quiescent nervous system and become enriched in the developing embryonic gonads, which will resume proliferation at larval hatching (Fig. 1D).

We identified embryonic neuroblasts (eNBs) by morphological criteria (Doe, 1992) and found that *ball* transcripts were enriched in most if not all eNBs that are arranged in a highly stereotyped pattern (Fig. 1E). Transcripts were less abundant in GMCs as shown by fluorescence *in situ* hybridization of *ball* transcripts combined with antibody staining for the GMC marker protein Prospero (PROS) (Fig. 1F–H). Anti-BALL antibody staining showed that also BALL protein was enriched in NBs (supplementary material Fig. S1). BALL protein is also present in GMCs (Fig. 1I–K), either due to expression of *ball* in GMCs or due to segregation of BALL to GMCs during eNB division, since BALL was associated with chromatin during mitosis (supplementary material Fig. S2). In summary, *ball* transcripts and protein are enriched but not exclusively present in embryonic neuroblasts.

In order to address the function of BALL for neuronal development, we used the *ball^2^* null allele (Herzig et al., 2014). Anti-BALL antibody staining revealed that *ball^2^* mutant eNBs have greatly reduced BALL levels due to the lack of zygotic *ball* expression (supplementary material Fig. S3). Such *ball^2^* mutant embryos were viable and hatched (98% of expected; n = 3037 embryos), and we detected no defects in their nervous systems based on staining with the 22C10 monoclonal antibody recognizing the *Futsch* protein (Fujita et al., 1982), which marks neurons of the central and peripheral nervous system (data not shown). Thus, BALL has either no critical function in eNBs or the level of maternally derived BALL protein in such embryos is sufficient to drive the apparently normal early embryonic nervous system formation in the absence of zygotic BALL expression.

### BALL is essential for larval brain development

**Fig. 2.**
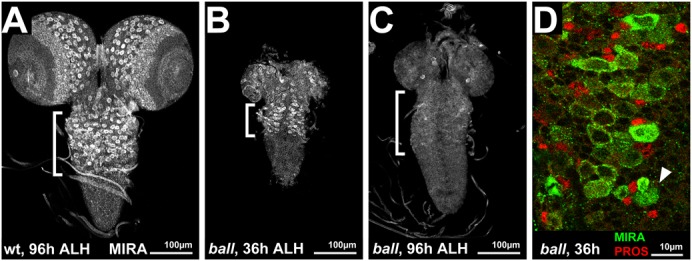
Miranda expressing pNBs are lost from *ball^2^* mutant larval brains. (A–C) Miranda (MIRA) expression detected by antibody staining of larval brains. The brackets indicate the position of thoracic pNBs in the ventral ganglion. (A) Wild type larval brains contain MIRA expressing pNBs at 96 h ALH. (B) *ball^2^* mutant brains carry MIRA expressing pNBs at 36 h ALH. (C) At 96 h ALH, *ball^2^* mutant brains lack MIRA expressing pNBs. (D) MIRA expressing pNBs of *ball^2^* mutant brains were initially functional, as evidenced by the asymmetric distribution of MIRA (arrowhead) and generation of Prospero (PROS) expressing GMCs. Scale bars in A–C, 100 µm. Scale bar in D, 10 µm.

The lethal phase of homozygous *ball^2^* mutants is the pupal stage (Herzig et al., 2014) (supplementary material Fig. S4). Prior to pupariation, mitotically active tissues of *ball^2^* mutant larvae, including the brain, were severely reduced in size, whereas no defects were observed in postmitotic endoreduplicating tissue of larvae (supplementary material Fig. S4). In the larval brains, mitotic proliferation depends on postembryonic neuroblasts (pNBs), which represent eNBs that re-entered proliferation after a phase of mitotic quiescence (Sousa-Nunes et al., 2010). Before the pNBs resume proliferation, they increase in cell size and express the NB marker protein Miranda (MIRA). In wild type larvae, large MIRA expressing pNBs are maintained until the end of larval development (Fig. 2A) and continue to express high levels of BALL (supplementary material Fig. S5). In *ball^2^* mutant brains, however, MIRA expressing pNBs were present in early but not in late stage larvae (Fig. 2B,C). pNBs of early *ball^2^* mutant larvae were dividing, as shown by the asymmetric distribution of MIRA during pNB division and by the presence of PROS expressing GMCs next to the pNBs (Fig. 2D). These observations suggest that BALL is not strictly required for cell cycle progression of pNBs but for their maintenance.

We therefore asked whether pNBs were lost from *ball^2^* mutant brains or failed to express MIRA at later stages. To address this question, we used the MARCM system in order to label pNBs independently of neuroblast markers (Lee and Luo, 2001). With this approach, individual *ball^2^* mutant pNBs were generated that express a *tub-GAL4*-driven *UAS-lacZ* (β-Galactosidase, β-Gal) reporter gene irrespective of their stem cell identity. This experimental design allowed us to find out whether *ball^2^* mutant pNBs were maintained at later stages of larval development and also whether they generated complete cell lineages. By focusing on thoracic pNBs of the ventral ganglion (Fig. 2), we determined the cell number in distinct cell lineages and the proliferation rate of a pNB.

**Fig. 3.**
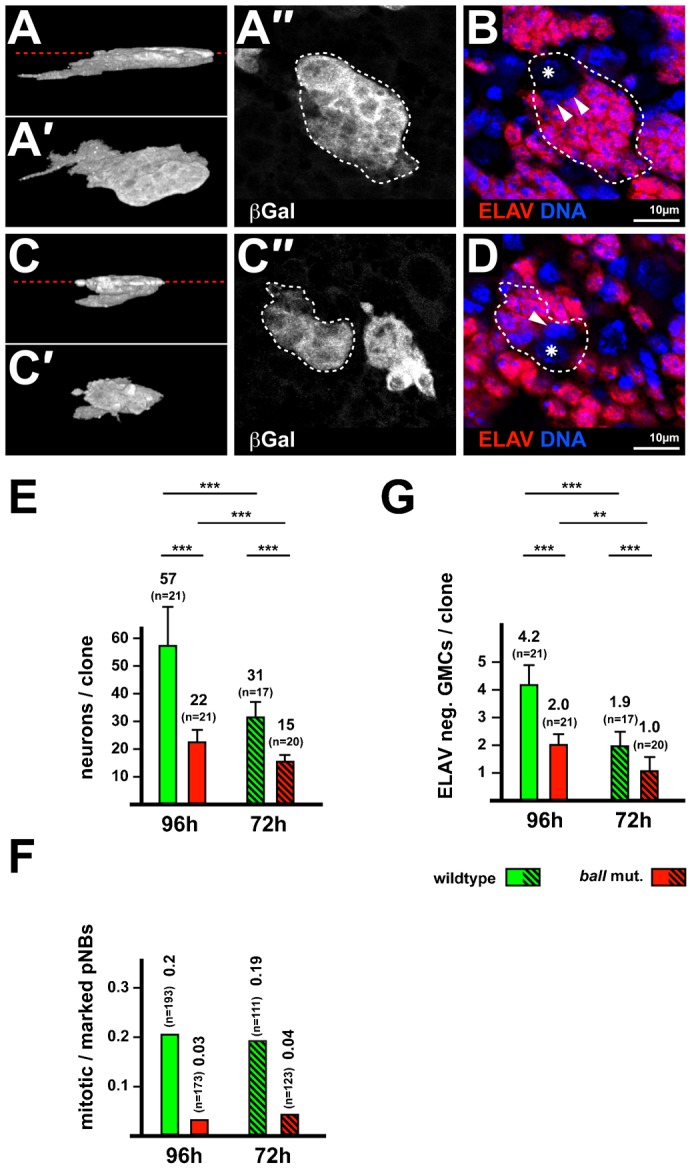
*ball^2^* mutant thoracic pNBs proliferate at a reduced rate. (A–A″) Control wild type cell lineage labeled by *tub-GAL4* dependent β-Galactosidase (β-Gal) expression through the MARCM system. (A) 3D reconstruction from confocal image sections. Also shown are tilted views of the reconstruction (A′) and a single focal plane (A″) of which the position is indicated by a red dashed line in (A). An individual cell lineage is marked by a white dashed outline. (B) Counterstaining for DNA and the neuronal marker Elav (ELAV). Asterisks indicate the position of the pNBs, arrowheads point at GMCs, which lack ELAV expression. (C–C″) *ball^2^* mutant cell lineages stained, analyzed and displayed the same way as control wild type lineages (A–A″). (D) Counterstaining as in (B). (E) Quantification of neuronal cell numbers in wild type control (green) and *ball^2^* mutant (red) cell lineages at 96 h and 72 h ALH. The Number of ELAV expressing cells per cell lineage is displayed on the y-axis (neurons). Mean values and the total number of cell lineages analyzed (n) are given above the bars. (F) Quantification of mitotic pNBs in wild type control (green) and *ball^2^* mutant (red) cell lineages at 96 h and 72 h ALH, respectively. The fraction of pNBs that were in mitosis, based on H3S10ph positive staining, is indicated on the y-axis. Mean values and the total number of marked pNBs (n) are given above the bars. (G) Quantification of GMCs lacking ELAV expression in wild type control (green) and *ball^2^* mutant (red) cell lineages at 96 h and 72 h ALH, respectively. The number of ELAV negative cells below pNB size per marked cell lineage is indicated on the y-axis. Mean values and the total number of cell lineages (n) are given above the bars. Significance levels from Student's t-tests: p <0.005 (**), p <0.0005 (***). Scale bar in A–D, 10 µm.

Thoracic pNBs resume proliferation at about 36 h after larval hatching (ALH) (Maurange and Gould, 2005). To visualize entire cell lineages that derived from wild type and *ball^2^* mutant NBs, we induced MARCM clones at 24 h ALH, dissected the brains at 96 h ALH and stained them with antibodies against β-Gal and the neuronal marker protein ELAV. Both wild type and *ball^2^* mutant lineages contained multiple ELAV positive neurons, small ELAV negative GMCs and one large ELAV negative pNB (Fig. 3), which was confirmed by antibody staining to visualize additional NB markers such as MIRA and aPKC (see below). The observation that *ball^2^* mutant pNBs were able to generate cell lineages including differentiating neurons demonstrates that BALL is dispensable for the differentiation of both GMCs and neurons.

### BALL regulates the rate of larval NB proliferation

Although BALL is not strictly required for cellular proliferation, we noticed a significant difference in the total volume of wild type (1,423.5 µm^3^, SD = 391.6 µm^3^, n = 30) and *ball*^2^ mutant cell lineages (862 µm^3^, SD = 254.2 µm^3^, n = 18) at 96 h ALH in the MARCM experiments. Anti-ELAV antibody staining revealed that the *ball*^2^ mutant cell lineages contained only about half the number of neurons (22.0 neurons, SD = 4.5, n = 21 lineages) than observed with the wild type controls (56.8 neurons, SD = 14.3, n = 21 lineages; Fig. 3E). This reduction of *ball*^2^ mutant neurons was rescued by re-expression of BALL through a *UASp-ball-EGFP* transgene in *ball*^2^ mutant lineages (40.2 neurons; SD = 6.9, n = 12 lineages), indicating that the *ball*^2^ mutant phenotype is indeed caused by the lack of BALL. It is important to note that 96% of the *ball^2^* mutant lineages at 72 h ALH (n = 227 lineages) contained a morphologically distinct pNB, although the lineages already were clearly reduced in size (Fig. 3E). This finding suggests that the reduced cell number in *ball^2^* mutant lineages is not caused by cell death of pNBs. In addition, we also immunostained larval brains at later stages with antibodies directed against activated Caspase 3, which is a marker for cell death (Xu et al., 2006), but could not detect an increased number of apoptotic cells in *ball*^2^ mutant cell lineages at 96 h ALH (n = 56 lineages). These results indicate that pNBs of *ball^2^* mutant have either a reduced rate of proliferation or they stopped proliferation after they have been marked by the MARCM system. To distinguish between these possibilities, we stained larval brains with antibodies directed against the mitotic marker histone H3S10ph at 96 h ALH. We found that *ball^2^* mutant pNBs were still dividing at 96 h ALH, but the number of mitotic pNBs was significantly lower than the number of mitotic control pNBs (Fig. 3F). This result indicates that the mutant pNBs did not cease proliferation. Thus, we asked whether the proliferation rate of pNBs was reduced. To address this question, we determined the increase in cell numbers of pNB lineages between 72 h and 96 h ALH. Although cell numbers increased in *ball^2^* mutant lineages between 72 h and 96 h ALH, this increase was only about one fourth of that in wild type lineages (Fig. 3E). To further rule out that the reduced number of neurons resulted from an accumulation of GMCs, we determined the numbers of GMCs in wild type and *ball^2^* mutant lineages. At 96 h or 72 h ALH, *ball^2^* mutant cell lineages contained about half the number of GMCs as compared to wild type control cell lineages (Fig. 3G). This result argues for a reduced rate of pNB proliferation in the absence of *ball* activity. We also asked whether raising the expression of *ball* in wild type pNBs causes an increase in the rate of pNB proliferation by over-expressing a *UASp*-*ball* transgene in wild type thoracic pNBs (Siegrist and Doe, 2005). To label the pNBs, we used the *worniu*-Gal4 driver to express the β-Gal marker either together with or, as a control, without the *ball* transgene. However, the percentage of mitotic *ball* over-expressing pNBs (17.6%; n = 728 pNBs) was not significantly different from mitotic pNBs expressing only β-Gal (19.8%; n = 758 pNBs). Together these results establish that BALL, although not essential for cell cycle progression *per se*, modulates the rate of pNB divisions and has a permissive function to maintain their specific proliferative potential.

### *ball* mutant pNBs fail to maintain neuroblast identity

**Fig. 4.**
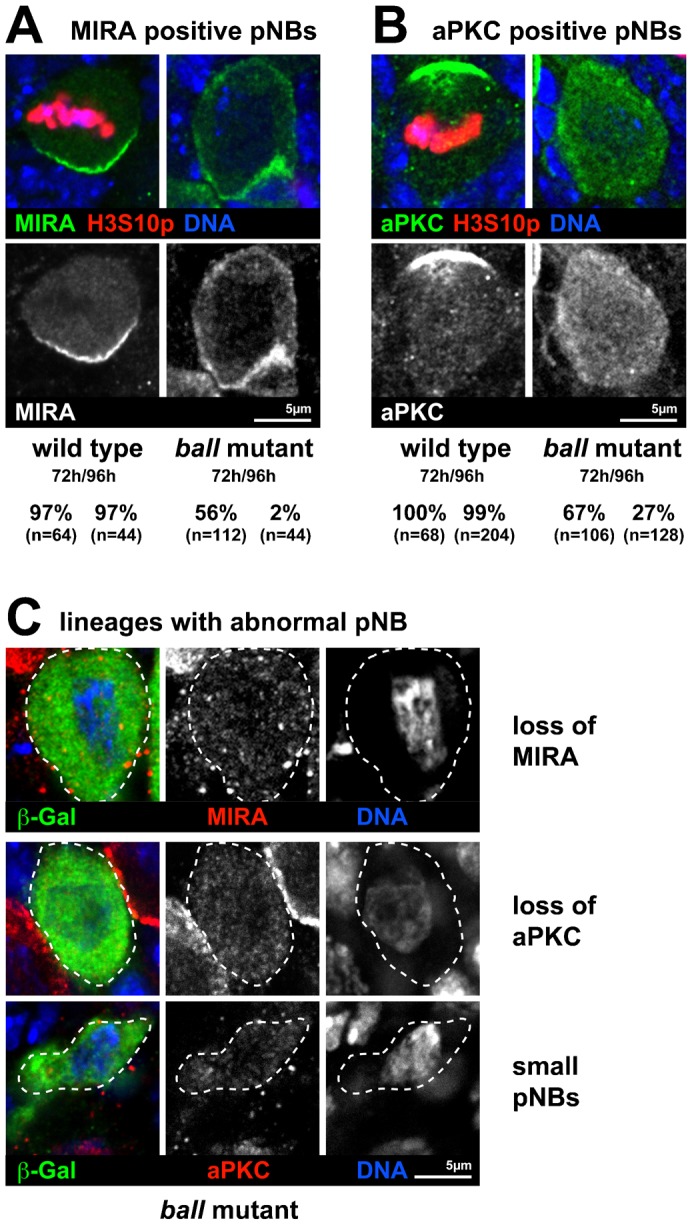
Untimely differentiation of *ball^2^* mutant thoracic pNBs. Wild type and *ball^2^* mutant cell lineages were labeled by *tub-GAL4* dependent β-Galactosidase (β-Gal) expression through the MARCM system. β-Gal staining is left out for clarity in (A,B). (A) Miranda (MIRA, green) localizes to the basal cortex of mitotic pNBs that express H3S10ph (red). In interphase pNBs, MIRA is cytoplasmic. DNA counterstain is shown in blue. The percentage of marked wild type pNBs that express MIRA at 72 h (97%) and 96 h (97%) ALH is indicated. The percentage of *ball^2^* mutant pNBs that express MIRA drops from 72 h (56%) to 96 h (2%) ALH. The total number of pNBs analyzed per time point (n) is indicated. (B) Atypical protein kinase C (aPKC, green) localizes to the apical cortex of mitotic pNBs which express H3S10ph (red). In interphase pNBs, aPKC is ubiquitous. DNA counterstain is shown in blue. The percentage of marked wild type pNBs that express aPKC at 72 h (100%) and 96 h (99%) ALH is indicated. The percentage of *ball^2^* mutant pNBs that express aPKC drops from 72 h (67%) to 96 h (27%) ALH. The total number of pNBs analyzed per time point (n) is indicated. (C) pNBs from β-Galactosidase (β-Gal, green) marked ball mutant lineages at 96 h ALH stained for Miranda (top panel, MIRA, red), atypical protein kinase C (lower panels, aPKC, red) and DNA (blue). The channels for MIRA/aPKC and DNA are also shown separately. The positions of pNBs are indicated by dashed outlines. In addition to MIRA/aPKC loss from the pNBs (note staining of neighboring non mutant pNBs), ball mutant pNBs were found to be reduced in size (small pNBs) and symmetric pNB divisions were observed. Scale bar, 5 µm.

Most of the *ball^2^* mutant MARCM lineages contained morphologically distinct pNBs at 72 h ALH (96%; n = 227). Since the brains of homozygous *ball^2^* mutant larvae lose expression of the pNB marker protein MIRA (Fig. 2), we also stained larval brains of MARCM experiments for MIRA, a determinant for pNB identity (Shen et al., 1997). We found that only about half of the *ball^2^* mutant pNBs expressed MIRA at 72 h ALH (56%; n = 112 pNBs; Fig. 4A). Furthermore, the apically localized pNB determinant aPKC (Wodarz et al., 2000) was expressed and properly localized in only about half of the *ball^2^* mutant pNBs (67%; n = 106 pNBs) (Fig. 4B) when compared with the respective control lineages (97%; n = 64 pNBs and 100%; n = 68 pNBs, respectively) (Fig. 4A,B). These data suggest that about half of the *ball^2^* mutant pNBs had lost neuroblast identity.

Niche-dependent *ball^2^* mutant germline stem cells (GSCs) (Herzig et al., 2014) undergo premature differentiation. Thus, we finally asked whether the loss of NB stem cell determinants results also in a premature differentiation of the *ball^2^* mutant pNB, i.e. both daughter cells develop into GMCs and subsequently into neurons. In wild type, thoracic pNBs undergo self-renewal until about 120 h ALH before they differentiate terminally into neurons (Maurange et al., 2008). This final differentiation step is characterized by a lengthening of the pNB cell cycle, loss of MIRA expression and a reduction of cell size (Maurange et al., 2008). As reported above, the proliferation rate of the *ball^2^* mutant NBs was reduced, implying a lengthening of the cell cycle, and MIRA expression was lost from about half of NBs. In addition, we found that the loss of NB determinants that we observed at 72 h ALH became progressively more severe till 96 h ALH. Amongst the *ball^2^* mutant pNBs that could be identified at 96 h ALH, only few expressed either MIRA (2%; n = 44 pNBs) or aPKC (27%; n = 141 pNBs), whereas nearly all of the control pNBs expressed MIRA (97%; n = 44 pNBs) and aPKC (99%; n = 204 pNBs) at the corresponding larval stage (Fig. 4A,B). Moreover, at 96 h ALH a significant number of the *ball^2^* mutant cell lineages contained no longer a morphologically distinct pNB (26%; n = 172 lineages) or a pNB with clearly reduced cell size (16%; n = 172 lineages), whereas time matched controls still contained a morphologically distinct pNB (97%; n = 250 lineages). Since these observations correspond to the events during the differentiation of wild type pNB, they suggest that *ball^2^* mutant pNBs differentiated prematurely between 72 and 96 h ALH.

## DISCUSSION

Our results establish that BALL is essential to maintain the proliferation rate as well as the undifferentiated state of pNBs and therefore interlink these two aspects of stem cell self-renewal. The proliferation rate of *ball^2^* mutant pNBs was reduced already at 72 h ALH, a time point when approximately half of the pNBs continued to express the stem cell determinants MIRA and aPKC. Therefore, it is plausible that the primary function of BALL is to control the proliferation rate of pNBs as a prerequisite for continuous self-renewal of neuroblasts.

The effects of a reduced proliferation rate were previously studied in epithelial tissue such as wing imaginal discs, which led to the discovery of a phenomenon termed cellular competition (Moreno et al., 2002). It describes that cells with reduced cellular fitness proliferate at a lower rate and are eventually eliminated by apoptosis. We observed this phenomenon after generating *ball^2^* mutant cells by MARCM in wing imaginal discs, showing that the mutant cells are capable to proliferate and to form cell clones. However, these cell clones fail to compete with wild type cells and subsequently undergo apoptosis (supplementary material Fig. S6). Maintenance of the stem cell character of pNBs is unlikely to be regulated through a competitive mechanism, since the pNB lineages contain only a single stem cell. Our data suggest that the same process that determines competitiveness of wing disc epithelial cells is a prerequisite to maintain the self-renewal of pNBs.

We have recently shown that BALL is required to sustain self-renewal of niche-controlled stem cells (Herzig et al., 2014). Here, we show that this function of BALL is not restricted to niche-controlled stem cells but is also required in pNBs, which depend on asymmetric distribution of cell fate determinants for self-renewal. Thus, the function of BALL for stem cell self-renewal is not limited by the factors and mechanisms that mediate cell fate decisions in the different stem cell systems. Our study therefore suggests that *Drosophila* stem cells employ cell intrinsic mechanisms to ensure stem cell self-renewal that are independent of the tissue specific modes of stem cell fate decisions and shared by diverse stem cell populations. The molecular basis of these mechanisms and how BALL is integrated in these processes remains to be established by future studies.

## Supplementary Material

Supplementary Material
