# Supplementary material for: Bällchen participates in proliferation control and prevents the differentiation of Drosophila melanogaster neuronal stem cells
Source: Biol Open. 2014 Sep 4;3(10):881–6. doi: 10.1242/bio.20148631 (PMC4197436; doi:10.1242/bio.20148631)
Supplement: Supplementary Material [file supp_3_10_881__index.html]

Bällchen participates in proliferation control and prevents the differentiation of Drosophila melanogaster neuronal stem cells — Bällchen participates in proliferation control and prevents the differentiation of Drosophila melanogaster neuronal stem cells — Supplementary Material 

# Bällchen participates in proliferation control and prevents the differentiation of *Drosophila melanogaster* neuronal stem cells

## bio.20148631 Supplementary Material

**Files in this Data Supplement:**

- Supplementary Material - Toma Yakulov et al. doi: 10.1242/bio.20148631
