# Supplementary material for: Bällchen participates in proliferation control and prevents the differentiation of Drosophila melanogaster neuronal stem cells
Source: Biol Open. 2014 Sep 4;3(10):881–6. doi: 10.1242/bio.20148631 (PMC4197436; doi:10.1242/bio.20148631)
Supplement: Supplementary Material [file supp_bio.20148631_bio.20148631-s1.pdf]

Biology Open (2014) 000, 1–6 doi:10.1242/bio.20148631

Supplementary Material

Toma Yakulov et al. doi: 10.1242/bio.20148631

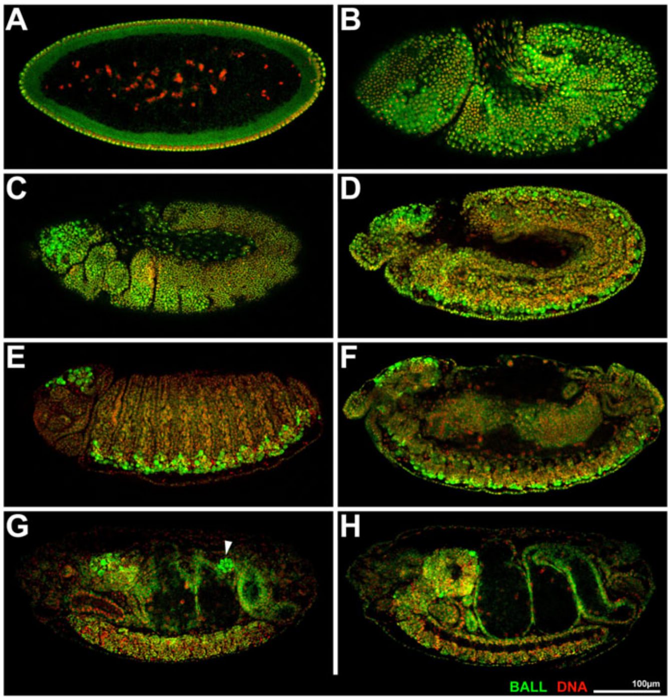

Fig. S1. BALL protein distribution in embryos. Embryos were stained with antibodies for BALL (green) and against DNA (red). The figures show optical sections from the interior (medial) or close to the epidermal layer (apical) of the embryo. BALL protein was ubiquitous till completion of the early cleavage divisions (A, medial) and subsequent stages (B, apical). During stage 12 of embryogenesis BALL is still present in the epidermis (C, apical) but becomes enriched in the nervous system (D, medial). BALL protein in the epidermal layer is likely persisting from earlier stages as *ball* mRNA was undetectable in this tissue by this stage (Fig. 1). Clear enrichment of BALL in the nervous system is observed by stage 13 (E, apical; F, medial). Note that BALL protein is highest in the peripheral layer of the nervous system that harbors the eNBs. Stage 17 embryos briefly before hatching into larvae (G,H) showed strong staining only in few cells of the nervous system and in the gonads (G, arrowhead). Scale bar, 100 µm.

Biology Open

Biology Open (2014) 000, 1–6 doi:10.1242/bio.20148631

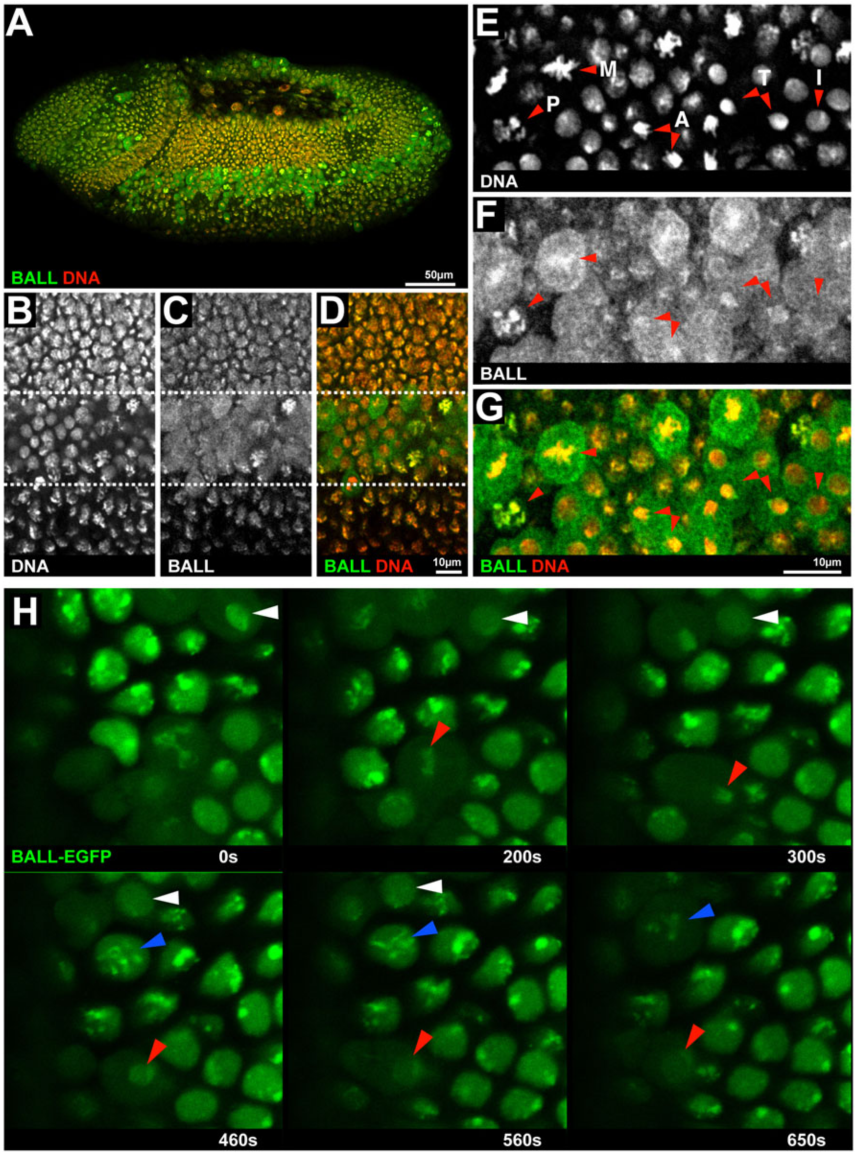

Fig. S2. BALL is associated with chromatin. (A) Whole mount embryo immunostained for BALL and counterstained for DNA. During early embryogenesis, BALL protein is found ubiquitously in epidermal tissue. (B–D) In interphase cells before mitosis 14 (below dotted lines) BALL co-localizes with DNA in the nucleus. During mitosis (between dotted lines) BALL is distributed throughout the cell and re-accumulates in the nucleus during the subsequent interphase of cell cycle 15 (above dotted lines). (E–G) In mitosis BALL co-localizes with chromosomes in the nucleus during prophase (P). During metaphase (M), anaphase (A) and telophase (T) BALL is found cytoplasmic as well as co-localized with DNA. During late telophase and early interphase (I) BALL is still found co-localizing with DNA, although it appears no longer to be enriched on chromatin. (H) BALL-EGFP fusion protein was expressed maternally by using *P{w^{+mC}=matalpha4-GAL-VP16}V3* maternal driver line. Embryos were collected, dechorionated and life embryos were imaged using a Leica SP2 LSM. Shown are selected images from a movie that show the dynamics of BALL-EGFP subcellular distribution in epidermal cells undergoing mitosis 14 during a 15 min time interval. Arrowheads are used to trace single nuclei. Chromosomal association of BALL-EGFP becomes obvious during prophase and a nucleoplasmic pool of BALL-EGFP appears to distribute into the cytoplasm after nuclear envelope breakdown (blue arrowheads, between 560 s and 650 s). Chromosomal association is visible in metaphase, anaphase and telophase (red arrowheads; 200 s, 300 s and 460 s, respectively). Clearance of BALL-EGFP from the cytoplasm is observed during early interphase (white arrowheads, 200 s to 560 s; starting from telophase at 0 s). Scale bar in A, 50 µm. Scale bar in B–D, 10 µm. Scale bar in E–F, 10 µm.

Biology Open

Biology Open (2014) 000, 1–6 doi:10.1242/bio.20148631

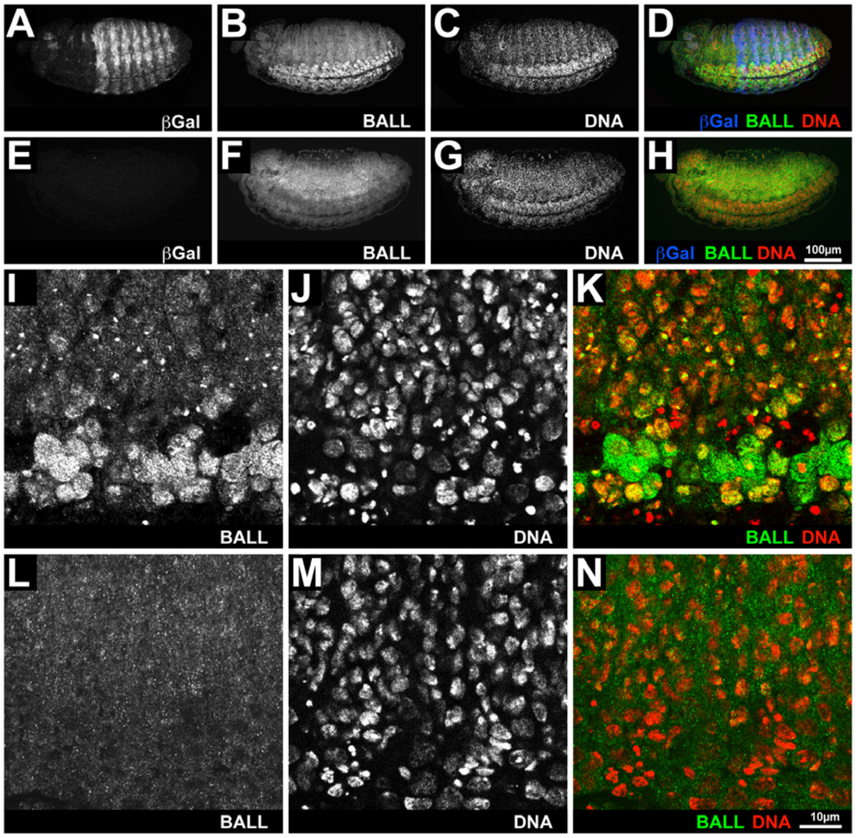

Fig. S3. BALL expression is strongly reduced in *ball*^{2} mutant eNBs. *ball*^{2} mutant and control sibling embryos were stained for β-Galactosidase (β-Gal, blue), BALL (green) and DNA (red). (A–D) Control embryos (β-Galactosidase positive) show strong BALL staining in the nervous system. (E–H) *ball*^{2} mutant embryos (β-Galactosidase negative) show no accumulation of BALL in the nervous system indicating that this expression domain resembles zygotic transcription of *ball*. (I–K) High magnification view of the nervous system in a wild type embryo. (L–N) High magnification view of the nervous system in a *ball*^{2} mutant embryo. Scale bar in A–H, 100 µm. Scale bar in I–N, 10 µm.

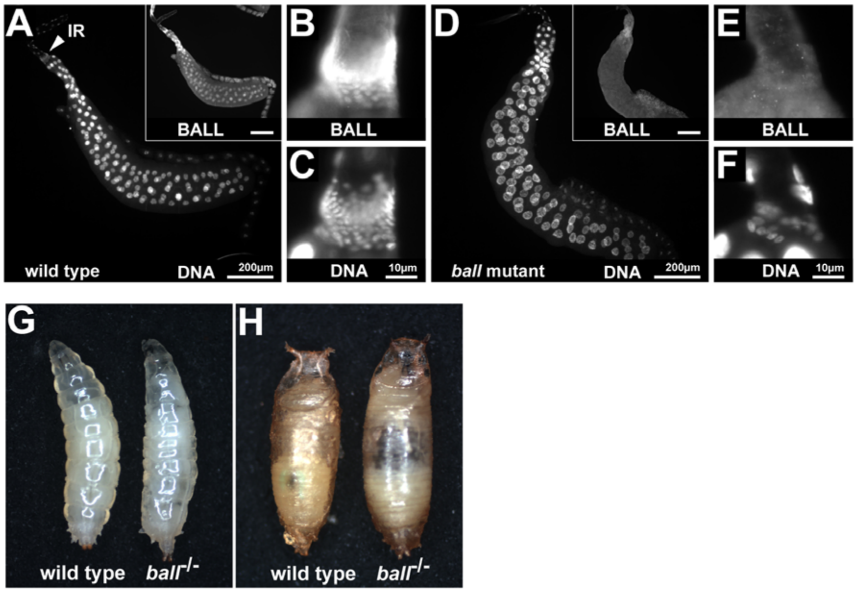

Fig. S4. BALL is not essential in endoreduplicating tissue. (A–C) Wild type larval salivary gland stained for DNA and BALL. In addition to large endoreduplicating and non-dividing cells the gland contains mitotically dividing cells in the imaginal ring (IR, arrowhead). BALL is expressed in endoreduplicating cells (inset, A) and in IR cells (B,C). (D–F) Endoreduplication of gland nuclei still occurs in *ball*^{2} mutant glands (D) although BALL is no longer detectable (D, inset). The proliferation of IR cells, however, is severely disrupted (E,F), which indicates that BALL is not essential for endoreduplication or S phase progression. (G) Wild type and *ball*^{2} mutant larvae grow to comparable size before pupariation. Since larval growth is largely accomplished by endoreduplication of differentiated tissue this supports the notion that BALL is largely dispensable in this process. (H) *ball*^{2} mutant larvae still form pupae but degenerate subsequently and die. Scale bar in A,D, 200 µm. Scale bar in B,C and E,F, 10 µm.

Biology Open

Biology Open (2014) 000, 1–6 doi:10.1242/bio.20148631

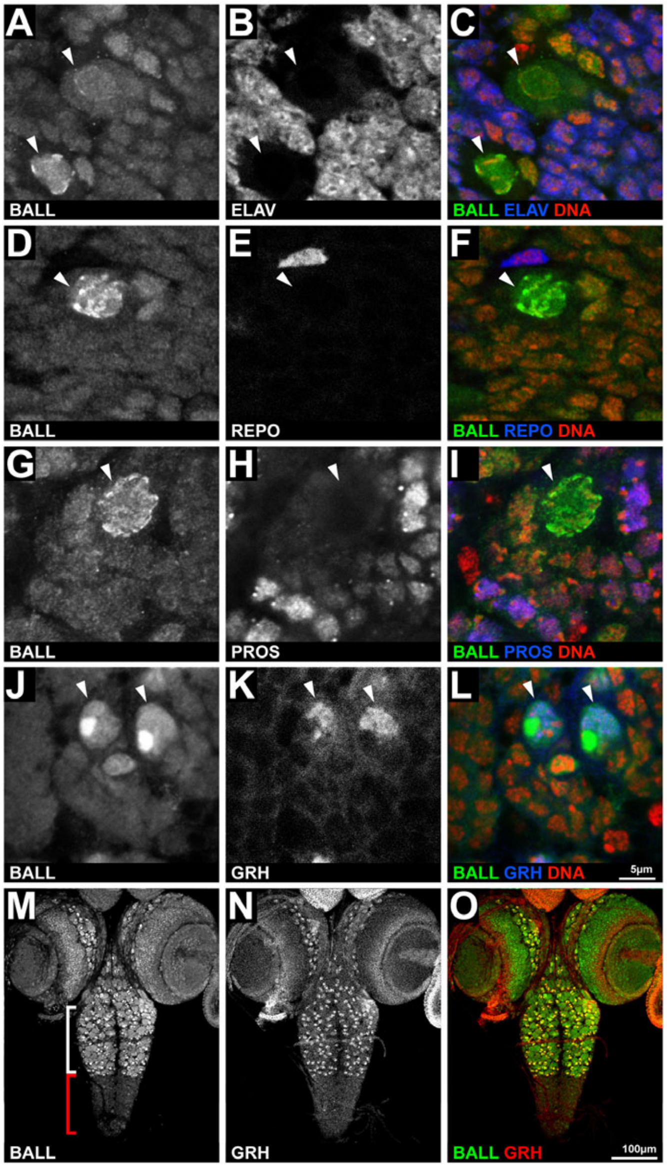

Fig. S5. BALL is enriched in thoracic pNBs. Wild type brains of larvae 96 h ALH were stained for BALL (green in overlays), DNA (red in overlays) and various marker proteins (blue in overlays). Neuroblasts (pNBs) of thoracic lineages are marked by arrowheads. (A–C) BALL levels are elevated in pNBs but BALL is also present in differentiating neurons that are marked by ELAV. (D–F) BALL is undetectable in glial cells that contact thoracic pNBs and are marked by the REPO protein. (G–I) In thoracic pNB lineages PROS is expressed in neurons that are born early during larval development. We could not detect a differential expression of BALL in PROS positive versus negative cells. (J–L) BALL expression in thoracic neuroblasts was verified by co-localization with the transcription factor Grainyhead (GRH). (M–O) Whole mount brain 96 h ALH stained for BALL (green) and GRH (red). pNBs of the abdominal region of the ventral ganglion (red bracket) only generate small lineages during larval development since they get eliminated by apoptosis. BALL levels in the abdominal region of the ventral ganglion are much lower than in the thoracic region (white bracket) that contains continuously proliferating pNBs. Scale bar in A–L, 5 µm. Scale bar in M–O, 100 µm.

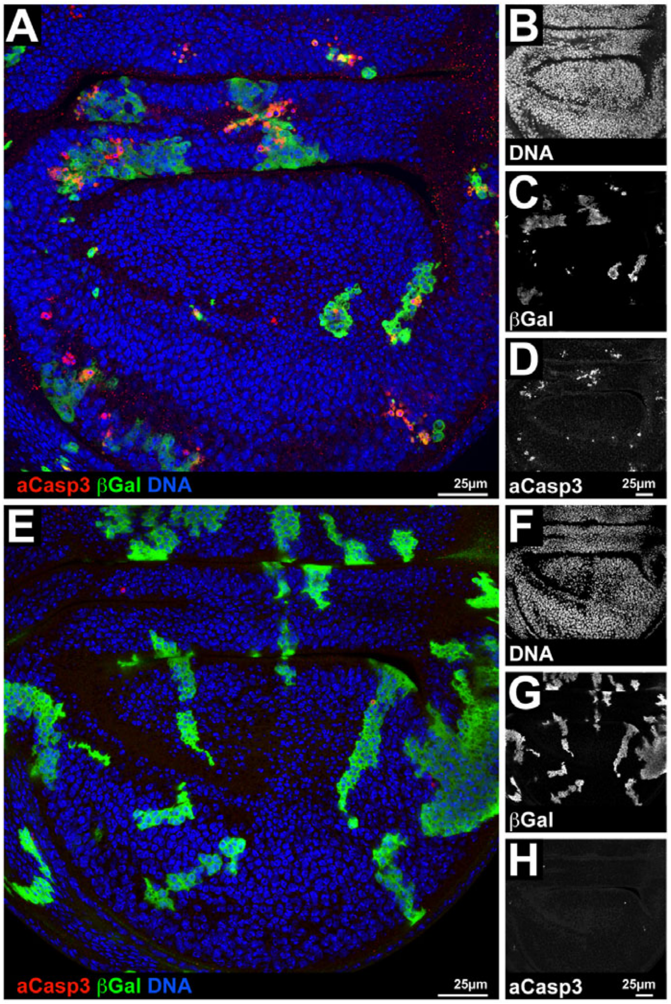

Fig. S6. *ball*^{2} mutant cell clones in wing imaginal discs are eliminated by apoptosis. (A–D) *ball*^{2} mutant MARCM clones were induced at 24 h ALH and wing discs were dissected at 96 h ALH. We observed positively β-Galactosidase marked cells (β-Gal, green in overlay) indicating that *ball*^{2} mutant cells can proliferate. However we found many cells in the clones stained for cleaved Caspase 3 (aCasp3, red in overlay), indicating that *ball*^{2} mutant cells are eliminated by apoptosis. Counterstaining for DNA is blue in overlay. (E–H) Non-mutant control MARCM clones were induced as in (A–D). Cell numbers were significantly higher in the control clones than in

Biology Open
